# Increased frequency of metabolic syndrome among Vietnamese women with early rheumatoid arthritis: a cross-sectional study

**DOI:** 10.1186/ar3203

**Published:** 2010-12-23

**Authors:** Hanh-Hung Dao, Quan-Trung Do, Junichi Sakamoto

**Affiliations:** 1 Department of Young Leaders' Program in HealthCare Administration, Nagoya University Graduate School of Medicine, 65 Tsurumai-cho, Nagoya 466-8550, Japan; 2 Rheumatology Division, Outpatient Department, Bach Mai University Hospital, 78 Giai Phong Avenue, Hanoi, Vietnam; 3 Endocrinology Division, Outpatient Department, Bach Mai University Hospital, 78 Giai Phong Avenue, Hanoi, Vietnam

## Abstract

**Introduction:**

Rheumatoid arthritis (RA) is associated with increased morbidity and mortality due to cardiovascular disease, and this occurs early in the disease process. The metabolic syndrome (MetS) may contribute to the excess cardiovascular burden observed in RA; however, little information is available regarding MetS in early RA. We aimed to identify the prevalence of MetS and to determine the potential factors associated with the presence of MetS in Vietnamese women with early RA.

**Methods:**

A total of 105 consecutive women with early RA (disease duration ≤3 years) and 105 age-matched healthy women were checked for MetS according to six MetS definitions (Joint Consensus, International Diabetes Federation, National Cholesterol Education Program 2004 and 2001, European Group for Study of Insulin Resistance, and World Health Organization). Multivariate logistic regression models were constructed to determine independent predictors of MetS in women with RA.

**Results:**

Prevalence of MetS varied from 16.2% to 40.9% according to the definitions used in women with RA, and was higher (*P *< 0.001) than in healthy controls (from 10.5% to 22.9%). Among individual components of MetS, differences between women with RA and controls were observed for hypertension (*P *< 0.001), low high density lipoprotein-cholesterol (HDL-C) levels (*P *< 0.001), and abdominal obesity (*P *= 0.019). After adjusting for age and physical activity, higher erythrocyte sedimentation rate (ESR) (odds ratios (OR) = 1.516, 95% confidence interval (CI): 1.073 to 3.195, *P *= 0.042), disease activity score (DAS28) (OR = 1.736, 95% CI: 1.293 to 2.786, *P *= 0.019), health assessment questionnaire (HAQ) score (OR = 1.583, 95% CI: 1.195 to 2.367, *P *= 0.035), and less methotrexate use (OR = 0.736, 95% CI: 0.547 to 0.962, *P *= 0.024) remained significant independent predictors of the presence of MetS in women with RA.

**Conclusions:**

Women with early RA already had higher prevalence of MetS compared with healthy controls. Higher systemic inflammatory marker, disease activity and disability scores, and less methotrexate use were independent predictors associated with the presence of MetS in women with early RA. These findings suggest that physicians should screen for MetS in women with early RA to control its components and therefore reduce their risk of cardiovascular diseases.

## Introduction

Rheumatoid arthritis (RA), the most common chronic inflammatory arthritis in women, is associated with increased morbidity and mortality [1] due to cardiovascular disease (CVD) [2], mostly accelerated atherosclerotic CVD [3,4]. Therefore, European League Against Rheumatism (EULAR) guidelines recommend that cardiovascular risk screening and management strategies are urgently needed in patients with RA [5]. Such strategies are generally done on the basis of a cardiovascular risk score calculator, such as the Framingham score (often used in the United States) [6] and the Systemic Coronary Risk Evaluation (SCORE) model (often used in Europe) [7]. In these models, traditional cardiovascular risk factors such as age, gender, smoking status, blood pressure (BP), cholesterol and high-density lipoprotein cholesterol (HDL-C) levels are integrated [5-8]. Risk estimates are based on information from the general population, however, little information regarding these models is available in RA populations [5,8].

Although traditional cardiovascular risk factors such as hypertension [2,9], central obesity [10,11], dyslipidaemia [12,13], and insulin resistance [14-16] may occur more frequently among patients with RA, this does not fully account for the rates of CVD observed [17], novel risk factors, particularly systemic inflammation, have also been implicated [18].

Metabolic syndrome (MetS), also known as syndrome X and insulin resistance syndrome, is a cluster of classical cardiovascular risk factors including insulin resistance, central obesity, hypertension, high triglycerides (TG) levels and low HDL levels [19]. MetS has been identified as an independent cardiovascular risk factor, conferring risk beyond the sum of its individual components [20]. MetS increases the risk for atherosclerotic CVD up to three times, and for type 2 diabetes mellitus up to five times [21]. Furthermore, MetS also increases mortality from CVD and all-causes in the general population [22]. At present, six definitions for MetS have been established: the Joint Consensus 2009 of the International Diabetes Federation (IDF) Task Force, National Heart, Lung, and Blood Institute, American Heart Association, World Heart Federation, International Atherosclerosis Society, and International Association for the Study of Obesity [23], the IDF 2005 [24], the National Cholesterol Education Program (NCEP) 2004 [21] and 2001 [25], the European Group for Study of Insulin Resistance (EGIR) 1999 [26], and the World Health Organization (WHO) 1998 [27]. These definitions have many similarities; however, they differ in some of the components, as well as in their specified cut-offs and weighting. In the general population, the prevalence of MetS has been shown to vary considerably according to the definition used, with the IDF criteria tending to report the highest and the EGIR criteria the lowest [23]. In patients with RA to date, eight other studies [28-35] and two reviews [36,37] have commented on the prevalence of MetS, reporting prevalence rates ranging from 12.1 to 45.3%, but most of the studies have been conducted in the long-standing disease (9.5 to 24 years). There is evidence that CVD morbidity and mortality occur early in the disease process [38,39]. Increased carotid intima media thickness [40-42], endothelial dysfunction [43,44], dyslipidaemia [45,46], and the pathogenic process for atherosclerosis may be in place even before a diagnosis of RA [43]. However, little information is available regarding MetS in early RA. Only one study [31] has investigated MetS in American patients with early RA (disease duration ≤3 years) and reported that the prevalence of the MetS was significantly greater in RA patients compared to controls.

There is evidence that under a given body mass index (BMI), body fat percentage is greater in Asians than Caucasians [47], and greater in RA patients than controls [48]. Therefore, Asian RA patients may be predisposed to more unfavourable cardiometabolic risk; however, there is no available information in the literature regarding MetS in this population.

During the last two decades, the socio-economic condition and lifestyle have profoundly changed in Vietnam; and these changes had strong effects on disease patterns in the population [49]. The prevalence of non-communicable diseases such as obesity, hypertension, and diabetes has been rapidly increasing; and the relationship among urbanization, sedentary lifestyle and these diseases was also demonstrated [49]. The mean BMI of Vietnamese increased from 19 to 23 kg/m^2^; and the prevalence of MetS recently reached 12% in the general population [50]. However, MetS has not yet been studied among patients with RA in Vietnam. Therefore, the present study was designed to (1) identify the prevalence of MetS according to all definitions currently used, in order to compare between other studies and (2) determine the potential factors associated with the presence of MetS in Vietnamese women with early RA.

## Materials and methods

### Study design and subjects

This study was designed as a cross-sectional investigation with two comparison groups. The first comprised 105 consecutive Vietnamese women with RA, from 26 to 73 years, who visited our Outpatient Department from October 2007 to March 2009. The second group was made up of 105 age-matched (± 2 years) healthy women who were selected randomly from applicants for an annual health check. They were judged normal on physical examination. All patients fulfilled the American College of Rheumatology (ACR) 1987 classification criteria for RA [51], with disease duration ≤3 years. Written informed consent based on the Helsinki Declaration was obtained from each subject. The study was approved by the Research and Ethical Review Board of the Bach Mai University Hospital, Hanoi, Vietnam.

### Assessments

Interviews were performed with a questionnaire identifying risk factors for MetS, such as lifestyle, age, smoking, menopausal status, disease duration and RA medications. A family history of coronary-artery disease was defined as a first-degree relative having had a myocardial infarction or stroke before age 55 in males and 65 in females [31]. Physical activity was defined by the seven-day physical activity recall questionnaire [52]. The assessments include a clinical examination, comprising swollen joint count (28 joints) and tender joint count (28 joints). Patients were also evaluated in terms of disease activity and disability using the disease activity score (DAS28) (using erythrocyte sedimentation rate (ESR) [53] and the Health Assessment Questionnaire (HAQ) [54], respectively. Pain and general health were measured by a visual analogue scale (VAS).

Height and weight were measured and BMI was calculated as body weight divided by the square of the height (kg/m²). In accordance with WHO standards, for Asian populations, individuals with a BMI <18.5 kg/m² are considered underweight, between 18.5 to 22.9 as normal, 23 to 27.49 as overweight and values greater than 27.5 indicate obesity [55]. Waist circumference (WC) was measured with an inelastic tape, placed directly on the skin, perpendicularly to the long axis of the body while the subject stood balanced on both feet, with both arms hanging freely. The measurement was taken at the end of expiration, at the midway between the costal arch and the iliac crest to the nearest 0.1 cm. BP was measured by a mercury sphygmomanometer in the sitting position after five minutes of rest.

Biological tests were performed from venous blood samples obtained the morning after an overnight fast. Plasma fasting glucose (FG) levels were measured using the glucose oxydase method. HDL-C and low-density lipoprotein cholesterol (LDL-C) levels were measured using corresponding non-precipitating method. Serum creatinine, TG, and total cholesterol (TC) were measured by an auto-analyser (Olympus AU 400, Olympus, Tokyo, Japan). A renal function assessment was performed by estimation of glomerular filtration rate according to the Modification of Diet in Renal Disease (MDRD) equation. For women with RA, rheumatoid factor (RF) and ESR were additionally measured. IgM-RF was assessed by enzyme-linked immunosorbent assay (ELISA), with seropositivity defined as ≥40 units.

The estimated cardiovascular risk of fatal CVD within 10 years was calculated using the SCORE model [7], according to the EULAR recommendations for cardiovascular risk management in patients with RA and other forms of inflammatory arthritis [5]. A cut-off point of SCORE >10% was used to define the subjects at high risk cardiovascular [5,7].

MetS was assessed according to all existing definitions (Joint Consensus [23], IDF [24], NCEP 2004 [21] and 2001 [25], EGIR [26], and WHO [27]). Details of these criteria are presented in Table 1.

**Table 1:** A summary of the definitions of the metabolic syndrome

	JC 2009	IDF 2005	NCEP 2004	NCEP 2001	EGIR 1999	WHO 1998
**Number of criteria**	Three or more of:	And two or more of:	Three or more of:	Three or more of:	And two or more of:	And two or more of:
**Obesity**	Population and country - specific definition	*WC ≥94 (men)* WC ≥80 (women)*	WC ≥102 (men) WC ≥88 (women)	WC ≥102 (men) WC ≥88 (women)	WC ≥94 (men) WC ≥80 (women)	BMI >30 and/or WHR >0.9 (men) WHR >0.85 (women)
**Hypertension (mmHg)**	≥130/85**	≥130/85**	≥130/85**	≥130/85**	≥140/90**	≥140/90
**HDL-C (mmol/l)**	< 1.0 (men) <1.3 (women)**	< 1.0 (men) <1.3 (women)**	< 1.0 (men) <1.3 (women)**	< 1.0 (men) <1.3 (women)**	< 1.0**	< 0.9 (men) <1.0 (women)**
**TG (mmol/l)**	≥1.7**	> 1.7**	≥1.7**	≥1.7**	> 2.0**	≥1.7**
**Glucose (mmol/l)**	≥5.6**	≥5.6**	≥5.6**	≥6.1**	≥6.1, *insulin in top 25%*	≥6.1, *DM, IGT, IR*
**Albumin/creatinine (mg/l)**	N/A	N/A	N/A	N/A	N/A	≥30

Text in italics: prerequisite for diagnosis, in addition to the number of other criteria needed to be met. * cut-off values differ according to ethnic origin, ** or treated for abnormality. BMI: body mass index; DM, diabetes mellitus; EGIR, European Group for Study of Insulin Resistance; HDL-C, high-density lipoprotein-cholesterol; IDF, International Diabetes Federation; IGT, impaired glucose tolerance; IR, insulin resistance; JC, Joint Consensus; N/A, not applicable; NCEP/ATP, National Cholesterol Education Program Adult Treatment Panel; TG, triglycerides; WC, waist circumference; WHO, World Health Organization; WHR, waist hip ratio.

### Statistical analyses

Data were presented as mean and 95% confidence interval (CI) for normally distributed continuous variables as well as median and inter-quartile range for skewed continuous variables. Frequency and percentage were used for categorical variables. Comparisons of the values between women with RA and controls were performed using the paired *t*-test for continuous variables and the chi-square test for categorical variables. Multivariate logistic regression models were constructed and odds ratios (OR) and 95% CI were calculated to investigate the independent of the predictors of individual RA-related characteristics and MetS in women with RA. All statistical analyses were done using the SPSS version 17.0 for Windows (SPSS Inc, Chicago, IL, USA). Statistical significance was defined as the two-tailed *P*-value < 0.05.

## Results

### Descriptive characteristics of study population

The median age was 56.3 and 55.7 years in women with RA and healthy controls, respectively. Women with RA had median disease duration of 21 months, and moderate disease activity (mean DAS28 score 4.1). The proportion of patients with low (DAS28 score <3.2), moderate (DAS28 score 3.2 to 5.1) and high (DAS28 score >5.1) disease activity were 36.2%, 52.5%, and 11.3%, respectively. The majority of patients with RA were currently treated with disease-modifying anti-rheumatic drugs (DMARDs) (89.5%), and glucocorticoids (68.6%) with mean daily dose of 8.6 ± 3.7 mg. Because biologic DMARDs have not yet been available in Vietnam, thus none of the patients with RA was treated with those drugs. There were no smokers among the participants.

Demographic and anthropometric characteristics of women with RA and healthy controls are presented in Table 2. No significant differences were seen between the two groups according to the proportion of postmenopausal female and family history of coronary disease. Compared with the healthy controls, women with RA had lower physical activity (*P *< 0.001). Although means of weight and BMI were similar between the two groups, the proportion of women with RA in the normal weight category was lower (*P *= 0.006), and in the overweight category was higher (*P *= 0.047) compared with healthy controls. WC was higher in women with RA compared with healthy controls (*P *= 0.007). Systolic BP was higher (*P *= 0.017) in women with RA while diastolic BP was similar between the two groups.

**Table 2:** Demographic and anthropometric characteristics of women with RA and healthy controls

Variables	RA patients (*n *= 105)	Controls (*n *= 105)	*P*-value
Age, median (range), years	56.3 (26 to 73)	55.7 (25 to 72)	0.583
Postmenopausal female, n (%)	52 (49.5)	49 (46.7)	0.394
Family history of coronary disease, n (%)	22 (20.9)	23 (21.9)	0.138
Regular intentional exercise, n (%)	26 (24.8)	43 (40.9)	< 0.001
Body weight, kg	54.7 (53.3 to 56.1)	53.4 (51.9 to 54.8)	0.369
Body mass index (BMI), kg/m^2^	23.1 (22.4 to 23.8)	22.5 (21.9 to 23.2)	0.473
Underweight (BMI <18.5), n (%)	12 (11.4)	9 (8.6)	0.296
Normal weight (BMI: 18.5 to 22.9), n (%)	41 (39.1)	52 (49.5)	0.006
Overweight (BMI: 23 to 27.49), n (%)	37 (35.2)	31 (29.5)	0.047
Obese (BMI ≥27.5), n (%)	15 (14.3)	13 (12.4)	0.317
Waist circumference, cm	85.3 (83.8 to 86.8)	78.5 (77.2 to 79.8)	0.007
Waist circumference ≥88, n (%)	16 (15.2)	9 (8.6)	0.005
Waist circumference ≥80, n (%)	49 (46.7)	32 (30.5)	0.019
Systolic blood pressure, mmHg	128.3 (126.1 to 130.5)	117.6 (115.5 to 119.7)	0.017
Diastolic blood pressure, mmHg	79.1 (77.9 to 80.3)	73.4 (72.3 to 74.5)	0.343
Blood pressure ≥130/85 mmHg, n (%)	39 (37.1)	27 (25.7)	< 0.001

Values are the mean (95% CI) unless otherwise indicated.

### Biological characteristics of study population

Means of total cholesterol and triglycerides levels were not significantly different between the two groups. As expected, HDL-C levels were lower (*P *= 0.018), and TC/HDL-C ratio and LDL-C were higher (*P *= 0.03 and 0.046, respectively) in women with RA compared with healthy controls. No significant differences were seen between the two groups according to glycaemia, creatinine, and creatinine clearance (Table 3).

**Table 3:** Biological characteristics and SCORE of women with RA and healthy controls

Variables	RA patients (*n *= 105)	Controls (*n *= 105)	*P*-value
Total cholesterol, mmol/l	5.3 (5.2 to 5.4)	5.2 (5.1 to 5.3)	0.296
Total cholesterol ≥5.2, n (%)	52 (49.5)	50 (47.6)	0.413
HDL-cholesterol, mmol/l	1.33 (1.29 to 1.37)	1.68 (1.62 to 1.74)	0.018
HDL-cholesterol ≤1.29, n (%)	53 (50.5)	24 (22.9)	< 0.001
Total cholesterol/HDL to C ratio	3.98 (3.77 to 4.19)	3.09 (2.86 to 3.32)	0.037
LDL-cholesterol, mmol/l	3.1 (2.9 to 3.2)	2.6 (2.5 to 2.7)	0.046
LDL-cholesterol ≥2.6, n (%)	49 (46.7)	48 (45.7)	0.161
Triglycerides, mmol/l	2.07 (2.04 to 2.11)	1.96 (1.94 to 1.98)	0.134
Triglycerides ≥1.69, n (%)	33 (31.4)	31 (29.5)	0.193
Glycemia, mmol/l	5.4 (5.3 to 5.5)	5.3 (5.2 to 5.4)	0.177
Glycemia ≥6.1, n (%)	10 (9.5)	9 (8.6)	0.219
Glycemia ≥5.6, n (%)	17 (16.2)	15 (14.3)	0.167
Creatinine, mmol/l	69.2 (67.9 to 70.4)	68.7 (67.5 to 69.9)	0.574
Creatinine clearance (ml/min)	80.8 (78.6 to 83.1)	81.6 (79.4 to 83.8)	0.358
SCORE function, %, mean (S.D.)	8.9 (3.6)	5.8 (2.7)	< 0.001
SCORE >10%, n (%)	16 (15.3)	9 (8.6)	< 0.001

Values are the mean (95% CI) unless otherwise indicated. HDL, high-density lipoprotein; LDL, low-density lipoprotein. RA, rheumatoid arthritis; SCORE, Systemic Coronary Risk Evaluation.

### Estimated 10-year cardiovascular risk of fatal CVD using the SCORE model

The SCORE function was higher (*P *< 0.001) and the proportion of high risk (SCORE >10%) in RA patients was almost doubled (15.3% vs 8.6%, *P *< 0.001) compared to those in healthy controls (Table 3).

### Prevalence of the metabolic syndrome in study population according to definition used

There was great diversity in the reported prevalence rates according to the definition used (Table 4). Prevalence of MetS in women with RA ranged from 16.2% to 40.9%, with EGIR reporting the lowest rate, the IDF reporting the highest rate, and the most updated Joint Consensus 2009 criteria and most commonly used NCEP 2004 reporting a rate of 32.4%. The prevalence rates were higher (*P *< 0.001) than that in healthy controls (ranging from 10.5% to 22.9%), and almost doubled in the young and middle-aged groups, irrespective of the criteria used. The prevalence increased with age (*P *< 0.001) in both groups (Table 4). Differences among women with RA and healthy controls were present for hypertension (*P *< 0.001), low HDL-C levels (*P *< 0.001), and abdominal obesity (*P *= 0.019). Among individual components of MetS, the most prevalent were low HDL-C levels, abdominal obesity, and hypertension in women with RA; and abdominal obesity, high TG levels, and hypertension in healthy controls (Tables 2 and 3). The proportion of high risk (SCORE >10%) was lower (*P *< 0.05) than prevalence of MetS, irrespective of the criteria used in the both women with RA and healthy controls (Tables 3 and 4).

**Table 4:** Prevalence of metabolic syndrome according to different criteria used

	n	JC 2009	IDF 2005	NCEP 2004	NCEP 2001	EGIR 1999	WHO 1998
**Total**							
**RA**	105	34 (32.4)†‡	43 (40.9)†‡	34 (32.4)†‡	26 (24.7)†‡	17 (16.2)†‡	20 (19.0†‡
**Controls**	105	19 (18.1)	24 (22.9)	19 (18.1)	15 (14.2)	11 (10.5)	13 (12.4)
**20 to 39 years**							
**RA**	19	5 (26.3)†	7 (36.8)†	5 (26.3)†	2 (10.6)†	1 (5.3)†	1 (5.3)†
**Controls**	19	2 (10.6)	3 (15.8)	2 (10.6)	1 (5.3)	0 (0)	0 (0)
**40 to 59 years**							
**RA**	51	16 (31.4)†	21 (41.2)†	16 (31.4)†	13 (25.5)†	8 (15.7)†	10 (19.6)†
**Controls**	51	8 (15.7)	10 (19.6)	8 (15.7)	7 (13.7)	5 (9.8)	6 (11.8)
**≥60 years**							
**RA**	35	13 (37.1)†	17 (48.6)†	13 (37.1)†	11 (31.4)†	8 (22.9)†	9 (25.7)†
**Controls**	35	9 (25.7)	11 (31.4)	9 (25.7)	7 (20.0)	6 (17.1)	7 (20.0)

Values are the number (%); EGIR, European Group for Study of Insulin Resistance; IDF, International Diabetes Federation; JC, Joint Consensus; NCEP/ATP, National Cholesterol Education Program Adult Treatment Panel; RA, rheumatoid arthritis; WHO, World Health Organization. †: p < 0.001 for comparison between RA patients and healthy controls; ‡: p < 0.001 for comparison between different age groups in RA patients and healthy controls.

### Associations of the metabolic syndrome in women with RA

Characteristics of women with RA who had and who did not have MetS are presented in Table 5. Results presented were only for the NCEP 2004 criteria, but were very similar when we used other criteria, despite the difference in prevalence.

**Table 5:** Characteristics of women with RA according to the presence or absence of metabolic syndrome

Variables	Total (*n *= 105)	With MetS (*n *= 34)	Without MetS (*n *= 71)	*P*-value
**Demographics**				
Age, median (range), years	52.7 (26 to 71)	54.6 (26 to 71)	50.8 (26 to 71)	0.003
Postmenopausal women, n (%)	52 (49.5)	16 (47.1)	36 (50.7)	0.271
Regular intentional exercise, n (%)	26 (24.8)	5 (14.3)	21 (29.6)	< 0.001
Family history of CHD, n (%)	22 (20.9)	7 (20.6)	15 (21.1)	0.537
**RA disease characteristics**				
RA duration, median (range), months	21 (3 to 36)	26 (3 to 36)	16 (3 to 36)	0.046
RF seropositivity, n (%)	73 (69.5)	25 (73.5)	48 (67.6)	0.049
ESR (mm in first hour), mean (S.D.)	27.5 (13.9)	33.6 (11.3)	21.4 (9.6)	0.038
DAS28 score, mean (S.D.)	4.1 (1.3)	4.7 (1.5)	3.5 (1.1)	0.007
HAQ score (range 0 to 3), mean (S.D.)	0.96 (0.57)	1.13 (0.58)	0.79 (0.55)	0.043
**SCORE function**, %, mean (S.D.)	8.9 (3.6)	9.7 (4.2)	8.1 (3.1)	< 0.001
SCORE >10%, n (%)	16 (15.3)	8 (23.6)	9 (12.6)	< 0.001

**Current RA medications**				
Methotrexate, n (%)	68 (64.8)	18 (52.9)	50 (70.4)	< 0.001
Sulphasalazine, n (%)	32 (30.5)	10 (29.4)	22 (30.9)	0.714
Hydroxychloroquine, n (%)	64 (60.9)	21 (61.7)	43 (60.5)	0.113
NSAIDs/COX-II, n (%)	29 (27.6)	10 (29.4)	19 (26.8)	0.139
Glucocorticoids use, n (%)	72 (68.6)	23 (67.6)	49 (69.1)	0.162
Anti-hypertensive, n (%)	20 (19.1)	9 (26.5)	11 (15.5)	< 0.001
Statin/fibrate, n (%)	19 (18.1)	11 (32.3)	8 (11.3)	< 0.001

CHD, coronary heart disease; COX-II, cyclooxygenase II inhibitor; DAS28, 28-joint disease activity score; ESR, erythrocyte sedimentation rate; HAQ, Health Assessment Questionnaire; MetS, metabolic syndrome; NSAIDs, non-steroidal anti-inflammatory drugs; RA, rheumatoid arthritis; RF, rheumatoid factor; SCORE, Systemic Coronary Risk Evaluation.

In univariate analysis, women with RA with the MetS were older (*P *= 0.003), had less regular intentional exercise (*P *< 0.001), longer disease duration (*P *= 0.046), higher RF positivity (*P *= 0.049), higher ESR (*P *= 0.038), higher DAS28 score (*P *= 0.007), higher HAQ score (*P *= 0.043), higher SCORE function (*P *< 0.001), higher proportion of SCORE >10% (*P *< 0.001), higher anti-hypertensive and statin/fibrate use (*P *< 0.001), and less methotrexate use (*P *< 0.001), compared with those who did not have the MetS. Sulphasalazine, hydroxychloroquine, glucocorticoids and NSAIDs/COX-II use were not significantly associated with the presence of the MetS. The independence of each of these associations was tested in a multivariate logistic regression model. After adjusting for age and physical activity, higher ESR (OR = 1.516, 95% CI: 1.073 to 3.195, *P *= 0.042), higher DAS28 score (OR = 1.736, 95% CI: 1.293 to 2.786, *P *= 0.019), higher HAQ score (OR = 1.583, 95% CI: 1.195 to 2.367, *P *= 0.035), and less methotrexate use (OR = 0.736, 95% CI: 0.547 to 0.962, *P *= 0.024) remained significant independent predictor of the presence of the MetS in women with RA (Table 6).

**Table 6:** Odds ratios for having the metabolic syndrome in women with RA*

Factors	Odds ratios (95% CI)	*P*-value
Disease duration	1.163 (0.971 to 1.924)	0.372
Rheumatoid factor seropositivity	1.092 (0.973 to 1.358)	0.547
ESR	1.516 (1.073 to 3.195)	0.042
DAS28 score	1.736 (1.293 to 2.786)	0.019
HAQ score	1.583 (1.195 to 2.367)	0.035
Methotrexate use	0.736 (0.547 to 0.962)	0.024

*Analyses are adjusted for age and physical activity. DAS28, 28-joint disease activity score; ESR, erythrocyte sedimentation rate; HAQ, Health Assessment Questionnaire; RA, rheumatoid arthritis.

## Discussion

This study was carried out in Vietnamese women with early RA and found that: 1. Prevalence of the MetS was significantly higher, almost doubled in the young and middle-aged groups, in women with RA compared with healthy controls, irrespective of the criteria used. 2. In women with RA, higher systemic inflammatory markers, or disease activity and disability scores, and less methotrexate use were independent predictors associated with the presence of the MetS, independently to age and physical activity.

To our knowledge, this is the first study to investigate the prevalence of MetS using all definitions in Asian patients with RA, and the second study in early RA in the literature [31]. Although most experts recognize that obesity-related insulin resistance may be the fundamental cause of MetS, each society has its emphasis in defining the syndrome. The WHO criteria [27] and the EGIR criteria [26] centre on diabetes and insulin resistance, whereas the IDF [24] focuses on central obesity as the essential condition, while the NCEP guidelines [21,25] give equal weight to each component of MetS like Joint Consensus 2009 [23]. Furthermore, cut-off points of individual components of MetS, particularly for WC, are different between the definitions (Table 1). This may explain a great diversity in the prevalence of MetS according to the definitions used, with EGIR reporting the lowest rate, the IDF criteria reporting the highest rate as shown in ours and an earlier study [32]. We used all MetS definitions currently used in order to compare our results to those of previous studies in patients with RA [28-35] and in the Vietnamese population [50]. The prevalence of MetS in women with early RA in our study was significantly higher than that in healthy controls, irrespective of the criteria used. These findings are in agreement with the results of earlier studies in both early RA [31] and long-standing RA [28-35].

In the literature, prevalence of MetS varied considerably, even using the same criteria; for example, using the NCEP 2001, the prevalence ranged from 17% in Mexican [30], 19% in South African [28], 19.9% in Dutch [34], 38.3% in English [32], to 41.5% in Swedish [33], 42% in American [31], and 44% in Greek [29] patients with RA. Such diversity can be explained by differences in the baseline characteristics and disease characteristics [28-34]. We found that women with RA had higher global estimated 10-year cardiovascular risk of fatal CVD using the SCORE model (based on EULAR guidelines) compared to healthy controls. These findings are in line with the earlier studies [8,56]. The differences between the rate of high risk CVD (SCORE above 10%) and MetS in our study were also reported in the general population using Framingham risk score [57]. The discrepancy may be explained by the fact that many individuals with MetS have borderline elevations in risk factors and thus may actually have either a low or intermediate risk of CVD [58]. It is thus important to determine one's 10-year cardiovascular risk in order to decide whether or not to start treatment [5,8]. In RA, treatment with statins and/or hypertensive agents should be started when the SCORE is above 10%, provided that the systolic BP is ≥140 mmHg and/or the LDL-C is ≥2.5 mmol/l [5]. It is noted that global risk scoring is heavily dependent on age and, therefore, underestimates risk of CVD in young individuals [57]. As MetS is not likely to replace currently used global risk scoring algorithms, both traditional risk factors and emerging metabolic markers associated with MetS should be incorporated in a future risk scoring system to be developed in order to adapt CVD risk prediction tools to the epidemic of obesity [58]. We found that among individual components of MetS, differences between women with RA and controls were observed for hypertension, low HDL-C levels, and abdominal obesity. These findings are consistent with earlier studies in early RA [31]. The prevalence of hypertension varied from 51.7% to 73% in patients with RA [9]. A consistent pattern of lower HDL-C levels is observed in patients with RA compared with age- and sex-matched controls [12,29,31,45,59] but there is conflict with regard to TC and LDL-C levels. We found TC levels were similar between the two groups while the atherogenic index (TC/HDL-C ratio) and LDL-C levels were higher in women with RA. These findings agree with an earlier study [45], but disagree with others [12,31]. Again, such discrepancy may be explained by differences in the baseline characteristics and disease characteristics.

In this study, although the mean of BMI was similar between the two groups, WC was higher in patients with RA compared with healthy controls. These findings agree with the results of earlier study in early RA [31]. The tendency towards abdominal obesity proves to be a better predictor than BMI of cardiovascular risk in the general population [21,24] and in RA [10,11]. The proportion of underweight, in both groups, was lower but overweight and obesity were higher than those in earlier population-based study in Hanoi [60], suggesting that although underweight remains the main concern, overweight and obesity make up an emerging burden in Vietnam.

The association between ESR and DAS28 score with the presence of the MetS in patients with RA in our study was also previously reported [29]. These findings further support the role of chronic inflammation in insulin resistance development [15]. Controlling systemic inflammation using anti-tumour necrosis factor (TNF) agents has been shown to lead to improvements in insulin resistance in patients with RA [16]. A higher HAQ score is likely to be associated with MetS in RA, because patients with more severe disabling disease are likely to lead a less active lifestyle, resulting in increased obesity and alterations in the lipid profile [61].

In this study, less methorexate use was associated with the presence of MetS in patients with RA. These findings agree with some earlier studies [30,32], but disagree with others [29,62]. These discrepancies may be explained by differences in the baseline characteristics and disease characteristics. Methotrexate use was associated with a reduction in CVD-related mortality [63] and improvements in lipid and glucose profiles, with lower TG levels, higher HDL-C levels and lower plasma glucose [32]. However, the mechanisms of action of methorexate are not clearly determined; this may be attributed to an anti-inflammatory effect [30] or a drug-specific effect [32]. Further investigations are needed to establish the effect of methotrexate on MetS.

No significant relationship between the presence of MetS and glucocorticoids use in this study was also previously reported [31,32]. Glucocorticoid use is associated with adverse lipid profiles in the general population, and its long-term use is a risk factor for CVD [64]. However, the relationship between glucocorticoid use and cardiovascular risk in patients with RA is complicated by the fact that these drugs tend to be used more often in patients with severe or intractable disease; therefore, it is difficult to determine whether the disease or the treatment increase the risk [64-66].

Prevalence of MetS in healthy controls was higher than that in an earlier study in Vietnam [50]. Furthermore, we also found that the prevalence of MetS increased with age in both groups as also reported in the earlier reports [31,32]. With the nation's increasing life expectancy, there will be a significant future increase in the prevalence of MetS. Therefore, weight and MetS control by association of dietary and physical activity enhancement should be emphasized for the prevention of obesity as well as the obesity-related CVD.

The results of this study must be interpreted within the limitations of the methods used. The major limitation was the small number of patients studied and that they were exclusively women. In practice, there were very few men with RA in our department, so we did not include them in this study; consequently, we are not be able to address the full scale problem of MetS in RA in Vietnamese patients. Previous study in the general population in Vietnam showed that men were more susceptible to Westernization through lifestyle modifications [21,50]. One study of 400 patients with RA reported that the prevalence of MetS was similar in men and women [32]; these findings differ from those observed in the general population [21,23-27], where age-matched men have been reported to have higher rates of MetS. This discrepancy may be a consequence of the ongoing inflammatory burden in the patients with RA, altering some of the components of MetS [32]. Further studies with larger patient cohorts with both men and women are useful. Also, we cannot exclude the possibility of patient selection bias, because our hospital is a tertiary referral centre. Another limitation was study design being cross-sectional, so it was not possible to make any cause-effect inference on the relationship between RA characteristics and MetS. Prospective studies should prove valuable in determining these causal relationships.

## Conclusions

Women with early RA already had higher traditional cardiovascular risk and prevalence of MetS compared with healthy controls. A higher systemic inflammatory marker, disease activity and disability scores, and less methotrexate use were independent predictors associated with the presence of MetS in women with early RA. These findings suggest that clinicians should screen for MetS in women with early RA to control its components and, therefore, reduce their risk of cardiovascular diseases.

## Abbreviations

ACR: American College of Rheumatology; BMI: body mass index; BP: blood pressure; CI: confidence interval; COX-II: cyclooxygenase II inhibitor; CRP: C-reactive protein; CVD: cardiovascular disease; DAS28: 28-joint disease activity score; DMARDs: disease modifying anti-rheumatic drugs; EGIR: European Group for Study of Insulin Resistance; ESR: erythrocyte sedimentation rate; FG: fasting glucose; HAQ: Health Assessment Questionnaire; HDL: high density lipoprotein-cholesterol; IDF: International Diabetes Federation; JC: Joint Consensus; LDL: low density lipoprotein; MetS: metabolic syndrome; MDRD: Modification of Diet in Renal Disease; NCEP/ATP: National Cholesterol Education Program Adult Treatment Panel; NSAIDs: non-steroidal anti-inflammatory drugs; OR: odds ratio; RA: rheumatoid arthritis; RF: rheumatoid factor; SCORE: Systemic Coronary Risk Evaluation; TC: total cholesterol; TG: triglycerides; TNF: tumor necrosis factor; VAS: visual analogue scale; WC: waist circumference; WHO: World Health Organization.

## Competing interests

The authors declare that they have no competing interests.

## Authors' contributions

HHD was responsible for the design of the study, for all measurements, for analyzing the data and for writing the draft manuscript. QTD was responsible for the design of the study and for revising the draft manuscript. JS made substantial contributions to analysis and to revision of the draft manuscript. All authors read and approved the final manuscript.
